# Surgeons' requirements for a surgical support system to improve laparoscopic access

**DOI:** 10.1186/s12893-022-01724-7

**Published:** 2022-07-19

**Authors:** Moritz Spiller, Marcus Bruennel, Victoria Grosse, Thomas Sühn, Nazila Esmaeili, Jessica Stockheim, Salmai Turial, Roland Croner, Axel Boese, Michael Friebe, Alfredo Illanes

**Affiliations:** 1 INKA-Innovation Laboratory for Image Guided Therapy (IGTLAB), Medical Faculty, Otto-von-Guericke-University Magdeburg, Magdeburg, Germany; 2 Otto-von-Guericke-University Magdeburg, Magdeburg, Germany; 3 Department of General, Visceral, Vascular and Transplantation Surgery, Medical Faculty, University Hospital Magdeburg, Magdeburg, Germany; 4 Department of Pediatric Surgery, Department of General, Visceral, Vascular and Transplantation Surgery, Medical Faculty, University Hospital Magdeburg, Magdeburg, Germany; 5 Department of Measurement and Electronics, AGH University of Science and Technology, Kraków, Poland

**Keywords:** Minimally invasive surgery, Laparoscopy, Laparoscopic access, Intraoperative support systems, Online questionnaire, Survey, Capnoperitoneum, Pneumoperitoneum, Peritoneal cavity, Audio sensing

## Abstract

Creating surgical access is a critical step in laparoscopic surgery. Surgeons have to insert a sharp instrument such as the Veress needle or a trocar into the patient’s abdomen until the peritoneal cavity is reached. They solely rely on their experience and distorted tactile feedback in that process, leading to a complication rate as high as 14% of all cases. Recent studies have shown the feasibility of surgical support systems that provide intraoperative feedback regarding the insertion process to improve laparoscopic access outcomes. However, to date, the surgeons’ requirements for such support systems remain unclear. This research article presents the results of an explorative study that aimed to acquire data about the information that helps surgeons improve laparoscopic access outcomes. The results indicate that feedback regarding the reaching of the peritoneal cavity is of significant importance and should be presented visually or acoustically. Finally, a solution should be straightforward and intuitive to use, should support or even improve the clinical workflow, but also cheap enough to facilitate its usage rate. While this study was tailored to laparoscopic access, its results also apply to other minimally invasive procedures.

**Supplementary Information:**

The online version contains supplementary material available at 10.1186/s12893-022-01724-7.

## Introduction

The first step in laparoscopic surgery is the creation of the surgical access. This is necessary to establish the so-called capnoperitoneum, which creates the field of vision and work for the subsequent procedure and provides safe space for inserting other medical instruments subsequently. To create the capnoperitoneum, the so-called Veress needle needs to be inserted into the peritoneal cavity. Once the instrument is placed accordingly, CO_2_ is insufflated into the peritoneal cavity.

However, surgeons act “blindly” while introducing the needle since they are not supported by any technical means. The surgeons have to rely on their sense of touch to guide the Veress needle. A decreasing resistance after passing through a tissue layer and an additional quiet “click” caused by the needle’s spring mechanism (+ a colored indicator in the case of single-use Veress needles) serves as a guide. The surgeons literally feel and count the different tissue layers when inserting the needle into the peritoneal cavity to orientate themselves. Additionally, especially single-use Veress needles provide a colored indicator that is pushed back by the spring mechanism, when a layer is passed. Due to this subjective and error-prone technique, between 30 and 50% of all complications in laparoscopic surgery occur during the creation of the laparoscopic access [1, 2].

Even though alternative methods such as the open laparoscopic access are available today, the Veress needle is still widely used to create the laparoscopic access and insufflate the CO_2_ [3–6]. Imaging and further potential technical support systems are often associated with a complex and time-expensive setup and are therefore not used in clinical practice.

Minor complications such as injuries to the urinary tract, abdominal wall, hematoma/bleeding or too early CO_2_ insufflation occur in up to 10% of surgeries [1, 7]. The correction of those complications prolongs the duration of surgery by up to 12% [8]. Severe complications like injuries to blood vessels, the intestine or other organs occur in up to 4% of cases [2, 9–11]. Among these, 8–17% [12, 13] of vascular injuries and 2.5–5% of intestinal injuries are fatal [14]. This is partly due to the fact that 30–50% of bowel injuries and 13–50% of vascular injuries go unnoticed during surgery, leading to life-threatening sepsis or peritonitis (5–15% mortality rate) [13, 15, 16].

Depending on the types of complications considered by studies in this area, the overall complication rate (mild + severe) when creating a laparoscopic access with the Veress needle is reported to be as high as 14% [1]. In addition, many studies report that the risk of laparoscopic surgical access is underestimated because many complications are not reported [3, 8, 15, 17–19].

To overcome the limitations of the Veress needle, medical device manufacturers and researchers have developed multiple alternatives to the Veress needle, for example optical trocars or needles and trocars with additional safety mechanisms.

Some of them are new, specialized instruments [20–22], e.g. with force sensors [22] embedded in their tip. However, this approach requires the development of an entirely new instrument and leads to a complex certification process. Other solutions like Veress needles with additional feedback modalities, e.g., LaparoLight (Buffalo Filter, Lancaster, NY, USA), are still based on the unreliable spring-loaded mechanism of the original Veress needle. Therefore, such instruments have not been introduced into the market, yet.

Optical trocars have been more successful. Those instruments directly visualize the abdominal wall layers as they are penetrated. However, those instruments have not proven to provide significantly more safety than the Veress needle. Bhoyrul et al. [23] found that 87% of the trocar-related complications reviewed in their study occurred despite using trocars with a ‘safety shield’. Twenty-six of the 408 reported injuries resulted in the patient’s death. Furthermore, the FDA (U.S. Food and Drug Administration) reported 79 serious complications such as major vascular injures or bowel perforations related to the use of two optical trocars (Visiport; United States Surgical, Norwalk, CT, USA; and Optiview; Ethicon Endo-Surgery, Cincinnati, OH, USA) [24]. However, only two of those complications were reported in the medical literature, confirming that complications in laparoscopic access are underreported (see above). Even though those studies do not allow calculation of the incidence of injuries in laparoscopic access, they indicated that ‘safety shields’, designed to protect intraabdominal organs and blood vessels, do not provide such protection. Consequently, the FDA prohibited to use the term ‘safety shields’ in product labeling of optical trocars [25].

In summary, despite the research and development conducted in this area, laparoscopic access is still associated with considerable risks for the patients and increased stress for surgeons. This highlights the need for a surgical support system to avoid the complications mentioned earlier during laparoscopic access, reduce the risk for patients and surgeons, and reduce the surgeons’ mental workload by simplifying the procedure.

Motivated by the shortcomings of optical trocars and other alternative methods for laparoscopic access that should improve safety, Proximal Audio Sensing has been developed [26, 27] and has demonstrated to be able to differentiate between Veress needle events in laparoscopic access [28]. The technique is based on acoustic emissions (AE) concerning the mechanical vibrations generated by the interactions between the needle’s tip and tissue. To acquire these AE, an audio-based sensing unit is mounted to the proximal end of a Veress needle. Thereby, the system can acquire information about tool-tissue interactions non-invasively from outside the body, one of the main advantages of this approach. Furthermore, the acquired audio signal is processed using advanced signal processing techniques and/ or Machine Learning algorithms to generate real-time feedback for surgeons.

However, to date, it remains unclear what the surgeons’ perspective on a surgical support system, based on Proximal Audio Sensing, is. So far, to our knowledge, no studies report which information surgeons require to guide the instrument more precisely and efficiently and how this information should be imparted to provide an added value to them.

In order to gather that information missing in the scientific literature, an explorative study among German and Austrian surgeons experienced in laparoscopic access was conducted and the results are reported in this article. Its findings provide specifics about the surgeons’ requirements for a surgical support system to improve precision, efficiency and safety during laparoscopic access. While this study was tailored to laparoscopic access, its findings can also be applied to many other surgical procedures.

## Materials and methods

The explorative study consisted of two main parts. At first, semi-structured interviews with ten surgeons were conducted to gather more information about the topic in general and the different types of users (often called ‘personas’) relevant in the context of laparoscopic access.

The acquired information was used to construct an online questionnaire. Using online questionnaires allowed to reach surgeons all across Germany and potentially Europe. The questionnaire was implemented using LimeSurvey (LimeSurvey GmbH, Hamburg, Germany). The link to the online questionnaire was distributed via multiple channels such as newsletter mailing lists of surgical (research) organizations (under careful considerations of the GDPR), Social Media and personal contact. Surgeons that participated during the semi-structured interviews were not excluded from the online questionnaire.

The questionnaire consisted of five main parts:

- Demographical information and career level.
- Experience in laparoscopic access and which tool the participant uses or used.
- Advantages/disadvantages of the used tool.
- How participants guide their instrument during laparoscopic access today.
- What information is required to make laparoscopic access more precise, efficient and safe and ways to present such information.

The online questionnaire was anonymous, self-administered and non-validated. The ethics committee of the University Clinic Magdeburg waived the need for ethical approval, since the questionnaire was conducted anonymously, and the participating individuals provided their informed consent prior to filling the questionnaire. The consent was obtained and recorded via the survey application.

The surgeons invited to participate in the online questionnaire were asked to fill the questionnaire within 2 months. After the expiration of these 2 months, the survey was terminated. Most questions were associated with a five-level Likert scale to scale the participants’ responses. The Likert scale was considered equidistant, and interval scaled. It offered the possibility to choose from two consenting, one neutral and two declining options. In the remainder of this article, the two consenting options will be summarized as “upper box”, while the two declining options will be referred to as “lower box”.

The influence of demographical data on other factors was analyzed using Pearson’s Chi-squared test. The questionnaire results were analyzed using SPSS (Version 27.0) and are described in the following.

## Results and discussion

Sixty-three surgeons participated in the online survey, of which 33 completed all survey questions. The incomplete submissions were not included in the analysis. Since the survey was also distributed via social media and third-party mailing lists, the response rate is unknown. The raw data obtained from this study is available as supplementary material at the end of this article (Additional file 1).

### Demographical data

Thirty-three surgeons of different ages, positions, and specialties completed the survey and were included in the data analysis. Two of the participants are working in Austria; the others are employed at surgical clinics from all over Germany. Twenty-six participants were male, while seven were female.

**Fig. 1:**
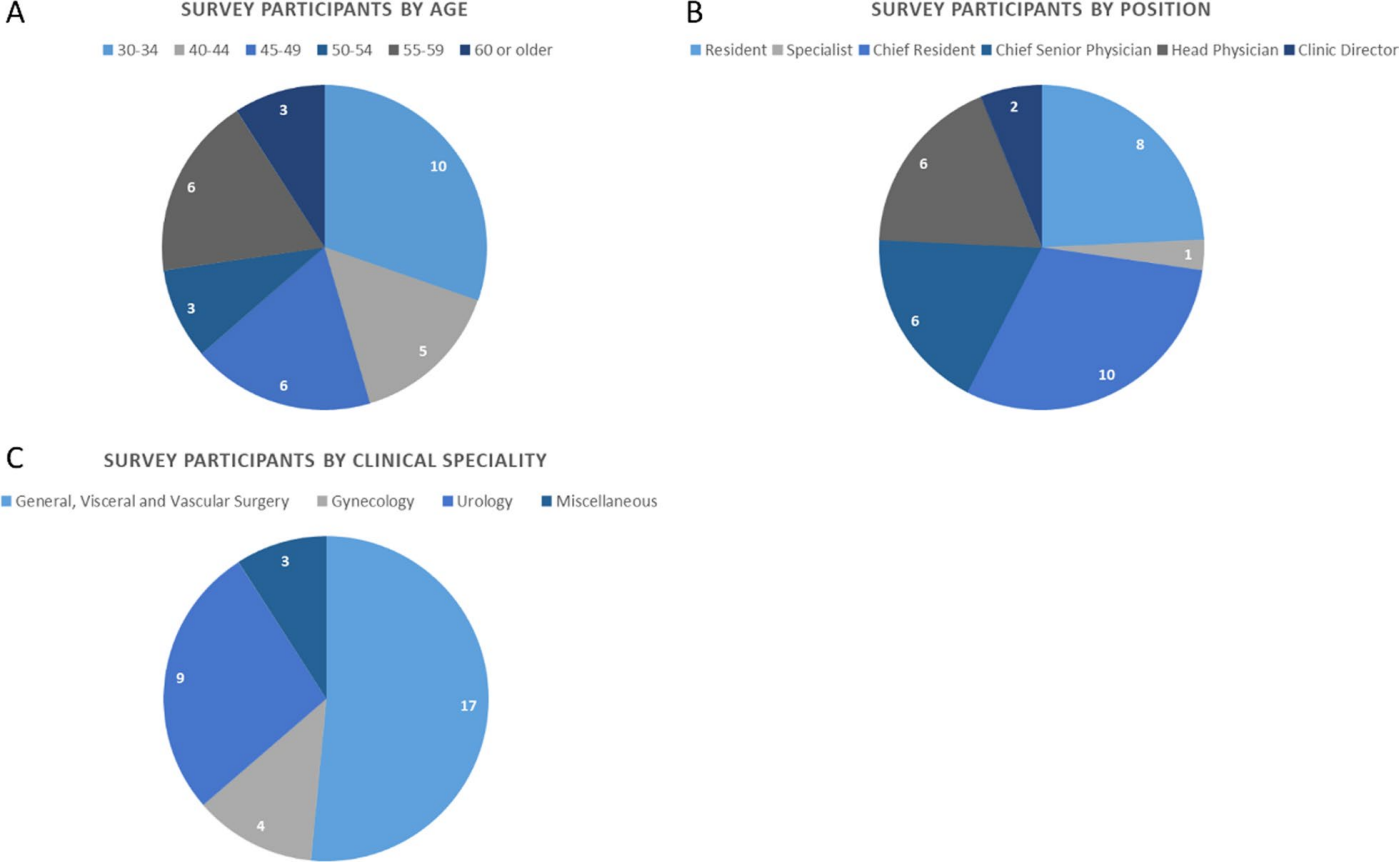
Demographical data of the survey participants by Age (**A**), Position (**B**) and Clinical Specialty (**C**)

Surgeons of all ages (see Fig. 1A) and clinical career levels (see Fig. 1B) participated in the survey. Most participants were Chief Residents or Residents (~ 55% of all participants) and were 30 to 54 years old (~ 82%). Options that did not apply to the participants were not included in the charts (e.g., the age group from 35 to 39).

The survey participants were asked to indicate the clinical specialty they are active in (see Fig. 1C). Most participants work in clinics for general, visceral and vascular surgery, followed by urologists and gynecologists. Three participants indicated to work in orthopedic and trauma surgery or anesthesia. However, also those participants indicated to have gathered experience in laparoscopic access earlier in their career.

Twenty of the participants voluntarily stated at which type of clinic they were employed. Five of those were employed at university clinics, the others at private (13) or public (2) hospitals.

### Instrument used for and experience in creating laparoscopic access

All participants stated that they have experience in performing laparoscopic access. Thirty of them are currently performing laparoscopic access in their everyday work, while three of the participants stated they are currently not involved in performing laparoscopic access. Those three participants are identical to the three surgeons who are working in Departments for orthopedic and trauma surgery or anesthesia (compare Fig. 1C (Miscellaneous)).

Twenty-six participants perform laparoscopic access multiple times per week, a procedure for which 22 participants use the Veress needle (67%). Eleven participants (33%) use alternative tools such as optical trocars, perform the procedure as a mini-laparotomy or use the open-access technique.

A Pearson’s Chi-squared test yielded no significant results. The participants’ age (Chi-squared(5, n = 33) = 7.05, p = 0.217), gender (Chi-squared(1, n = 33) = 0.363, p = 0.547) and their position (Chi-squared(5, n = 33) = 3.113, p = 0.683) did not have a significant influence on their choice of the Veress needle or an alternative tool.

### Advantages of the used instrument

Question 5 was intended to find out why a study participant is using the Veress needle or an alternative tool.

Most Veress needle users indicate using the Veress needle because they were trained to use it during their specialist training (90.9% of the Veress needle users) or because the Veress needle is the preferred tool at the clinic where they work (86.4%). Similar results can be observed for users of alternative instruments. Since 75% of the study participants had already finished their specialist training, surgeons seem to stick to the instrument they were trained on later in their careers.

95.5% of the Veress needle users see advantages in reduced time or preparation efforts. This is consistent with the users’ answers of the alternative tools and methods, where 45% and 36% of the participants indicated to perceive advantages in time and/or preparation efforts. Twenty-one of the 22 Veress needle users indicated an advantageous setup and use of the Veress needle. On the other hand, six of the 11 users of the alternative tools only indicated this, which could be another reason why the Veress needle is widely used despite its issues regarding safety.

36% of the participants do not feel safe and perceive a risk for injuring the patient while inserting the Veress needle. 55% state, that the feedback of the needle’s spring mechanism is not clear, especially regarding the reaching of the peritoneal cavity. Especially young surgeons (i.e., residents) were among them, indicating that users with minor experience feel uneasy and discomfort during the insertion of the Veress needle. This may lead to increased medical errors [29–31]. However, also five surgeons (Chief Residents and Chief Senior Physicians) at later career stages indicated to not feel safe and perceive a high risk for punctures during Veress needle insertion. This might also be caused by the ambiguous feedback provided by the spring mechanism of the Veress needle. 55% of he participants stated that they do not rely on the spring mechanism’s feedback. For the alternative methods, eight of the 11 participants reported that the feedback was clear, and only three stated it was not. Similar answers were provided to the question if the instruments’ feedback about the reaching of the peritoneal cavity was clear. Nine Veress needle users were undecided; three stated it is not clear and only ten (45%) feel that the needle’s feedback mechanism clearly indicates when the peritoneal cavity is reached. Ten out of 11 users of an alternative method stated that the information about the reaching of the peritoneal cavity is clear.

This highlights a significant problem of the Veress needle, which makes the procedure more complex: the primary goal of the procedure, the insertion of the Veress needle into the peritoneal cavity, is not clearly and reliably recognizable by the surgeons.

To gather data about the time expenditure for creating laparoscopic access, the study participants were asked to estimate the duration of the procedure with their instrument in three different cases (ideal, normal, worst) and how often those cases occur. The findings are summarized in Table 1.

**Table 1:** Summary of the participant’s estimations regarding time expenditure when creating laparoscopic access with the Veress needle or alternative tools such as trocars

	Veress needle		Alternative method	
	Duration [mm:ss]	Occurrence [%]	Duration [mm:ss]	Occurrence [%]
Ideal case	02:36	37.5	02:12	43.2
Normal case	03:36	51.4	04:30	45.9
Worst case	06:53	11.1	07:30	10.9
Average	04:22		04:44	

The findings show that the time expenditure for creating laparoscopic access in the ideal case is similar regardless of the used technique. In the average case, the use of the Veress needle shortens the duration of the procedure by almost 1 min. An advantage which diminishes in the worst case, where the participants stated a time expenditure increased by almost 100% (Veress needle) and 66% (alternative methods), respectively. In summary, Veress needle insertion is slightly quicker than alternative techniques such as the insertion of trocars. To increase the acceptance of a surgical support system for laparoscopic access, it should aim to enable its users to create surgical access in the duration that is required in the ideal case today (i.e., ~ 2.5 min).

### What helps surgeons to guide their instrument today

When guiding the instrument during laparoscopic access, surgeons can use different kinds of indications and/or feedback provided by the used instrument. As described in the introduction, every Veress needle has a spring mechanism that provides, depending on the specific manufacturer and type, visual and/ or acoustic feedback. In most Veress needles, the spring mechanism causes a “click” sound after passing through a tissue layer (acoustic feedback). Additionally, some Veress needles provide a colored indicator pushed back by the spring mechanism when a layer is passed (visual feedback). Whether or not the surgeons clearly perceive those feedbacks during the procedure remains unclear. On the other hand, optical trocars provide vision through a hollow inner core during insertion. In this case, only visual feedback is provided.

Independently from the used instrument, surgeons can feel a decreasing resistance after passing through a tissue layer. By counting the tissue layers passed and putting that into context with their anatomical knowledge, they can estimate in which tissue layer the instrument’s tip currently is (tactile feedback).

Despite the feedback provided by the instruments used and the tactile feedback that most surgeons perceive, the practical experience of each individual surgeon plays a decisive role in performing a safe and efficient Veress needle insertion.

**Fig. 2:**
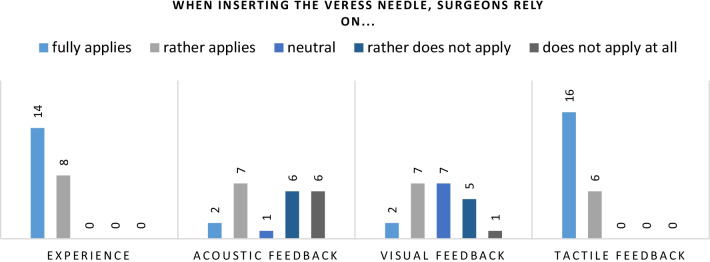
When inserting the Veress needle, surgeons rely heavily on practical experience and tactile perception. The “click” sound and the visual indicator, which are triggered by the needle’s spring mechanism, are not widely used

**Fig. 3:**
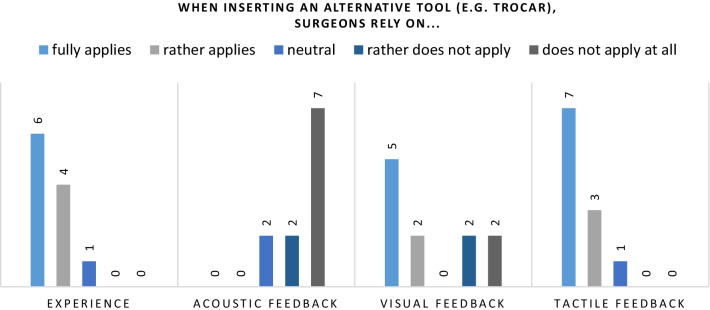
Similar observations like for the Veress needle could be made for alternative tools such as trocars. Most surgeons also rely on practical experience and tactile perception. The optical core (visual feedback), integrated as a safety mechanism into some trocars, is not used by everybody

To determine on which of the factors mentioned above the surgeons rely most during creating laparoscopic access, the study’s participants were asked to indicate to which extent they use each of them (see Figs. 2 and 3).

Both user groups rely heavily on their practical experience and tactile perception when creating laparoscopic access. 100% of the Veress needle users rely on experience. However, all residents in that user group chose “partly applies”, indicating that none of them relies on experience to a major extent. Only 41% of the Veress needle users rely on the acoustic feedback provided by the needle, i.e. the “click” caused by the needles spring mechanism, and the visual feedback caused by the colored indicator. Again, 100% of the Veress needle users rely on tactile perception during needle insertion.

Ten out of eleven surgeons using alternative methods stated to rely on experience and tactile perception as well. None of them relies on acoustic feedback, which is not surprising considering that none of their tools provides that kind of feedback. However, only seven out of 11 (63%) rely on the visual feedback that is provided by, e.g., optical trocars. That indicates that not all surgeons use this added feedback, which is meant to provide increased safety compared to the Veress needle. Instead, they still heavily rely on experience and tactile perception.

### What is needed to improve laparoscopic access

The next block of questions focused on the information that a surgical support system should provide for making laparoscopic access more precise, effective and safer. Additionally, the study participants were asked to indicate which peripherals (external devices, devices embedded into the sensing system) and sensory channels (visual, acoustic, tactile) they prefer to be used for imparting the system’s feedback.

The first question of this block consisted of a description of a surgical support system as described in “Introduction” section. Subsequently, the participants were presented a number of information that the surgical support system could potentially provide to them and rate those information according to their relevance. The options the surgeons were asked to rate were:

1. The force that is necessary to insert the instrument into the peritoneal cavity.
2. How deep the needle was inserted already.
3. Which tissue layer is currently penetrated.
4. The number of tissue layers the needle has already passed.
5. The information whether or not the peritoneal cavity has already been reached.
6. The information if an intraabdominal structure has been punctured and potentially injured after the peritoneal cavity has been reached.

**Fig. 4:**
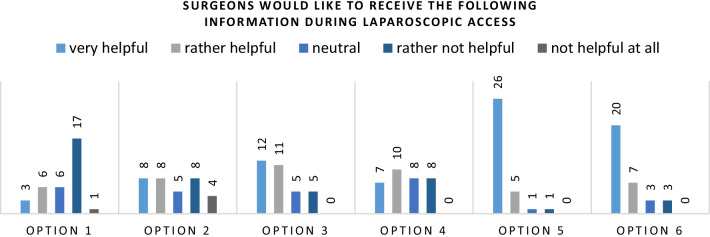
More than 60% of the participants would like to be supported by feedback regarding the reaching of the peritoneal cavity (option 5) and an alarm concerning a potential injury of intraabdominal structures (option 6) during laparoscopic access

The results are indicated in Fig. 4.

The information about the force that is necessary to insert the needle further was considered the least relevant information by the surgeons (18 participants in the lower box). The participants were divided over the relevance of the information about the depth of the needle and the number of penetrated tissue layers. The depth of the needle was relevant to 16 participants, while the number of penetrated tissue layers was relevant to 17 of them. The information about the reaching of the peritoneal cavity has been voted the most relevant information to surgeons during laparoscopic access, with 31 votes (94%) in the upper box. 26 of those even indicated this information to be “very helpful”, the highest rating possible. Almost the same number, 27 participants stated that the information about a potential injury is relevant (82%).

This clear result indicates that surgeons focus on two main aspects when creating laparoscopic access and successfully establishing the capnoperitoneum: (1) Is the peritoneal cavity already reached? and (2) Was any intraabdominal structure injured? A surgical support system based on proximal audio sensing should provide such information or make it easier for the surgeons to answer those questions themselves, to be accepted and perceived valuable to the users.

An increasing number of devices such as anesthesia machines, vital signs monitors, or endoscopy towers are used during surgery today. A surgical support system based on proximal audio sensing needs to facilitate the workflow of the surgeons and, therefore, not interfere with any of the other devices used in the operating room. Additionally, it should not take too much additional space in the already packed OR (Operating Room) and should be easy to use. However, there are multiple options to display the feedback using either the sensing unit or external devices. Because of this, the participants were asked to rate the suitability of various options to impart the feedback.

Those options were:

1. Visual feedback via an external monitor in the OR.
2. Visual feedback via LEDs or a small display integrated into the sensing module.
3. Visual feedback via LEDs or a small display mounted onto the forearm of the surgeon.
4. Visual feedback via an Augmented Reality Headset.
5. Acoustic feedback via external speakers in the OR.
6. Acoustic feedback via a speaker integrated into the sensing module.
7. Acoustic feedback via a headset worn by the surgeon.
8. Tactile feedback via a vibration of the sensing module.
9. Tactile feedback via a vibration of a wearable device worn by the surgeon.

**Fig. 5:**
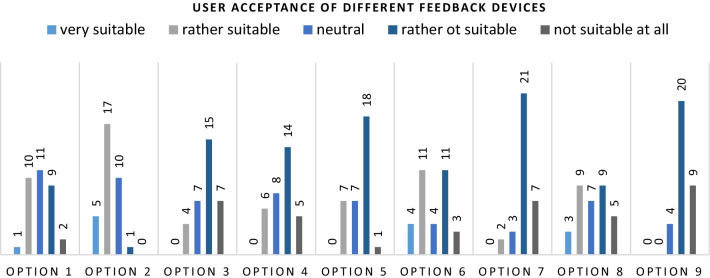
Most proposed feedback approaches were rejected by the participants. They mainly accepted feedback in their focus such as visual feedback via LEDs (option 2) or a speaker (option 6) integrated into the sensing module. Some surgeons can imagine the feedback to be displayed on an external screen (option 1) or as a vibration of the sensing module (option 8)

The results are illustrated in Fig. 5.

The options 3, 4, 5, 7 and 9 were not rated as suitable by a vast majority of the participants. Since the options 3, 4, 7 and 9 included wearable devices, this also shows that the acceptance of such devices during surgery is still low, at least for steps such as laparoscopic access. However, this could also be due to concerns about the impact of such devices on sterility of the protective gear and further equipment. Since the noise exposure in the OR is already quite high, it is not surprising that surgeons also rated external speakers (option 5) as not suitable.

Options 1 (visual feedback via external monitor) and 8 (tactile feedback via vibration on sensing module) reached average approval ratings. 12 participants indicated that a vibrating sensing module would be suitable for imparting information about the insertion process, while 14 found this approach not suitable. While especially young participants might be used to tactile display via vibrations from e.g., their smartphones, this approach might have also raised concerns about the patient safety, since the vibration emitted from the sensing module would propagate via the instrument to the patient’s body.

The votes for option 1 were equally distributed between upper box, neutral position and lower box (11 votes each). Since the participants are most likely experienced in laparoscopy, they are used to guiding the surgical instruments while looking at the screen of the endoscopy tower, which could drive the participants voting in favor of the external screen. On the other hand, surgeons usually monitor the response of the tissue and the needle’s spring mechanism during laparoscopic access. A principle that most of the participants want to stick to but would be hampered using an external screen for imparting the feedback.

Being able to monitor the needle insertion process might also be the reason to prefer the visual feedback via LEDs integrated into the sensing module (option 2). Twenty-two participants voted in favor of this option, while only one indicated that this would not be suitable. Ten participants chose the neutral option. This feedback variant would also provide all the functionality necessary to indicate the surgeons about the reaching of the peritoneal cavity and a potential injury of intraabdominal organs. The most relevant information surgeons require during laparoscopic access (see above).

The last block of questions was intended to gather information about which specific feedback principle the surgeons perceive to impart the feedback information most intuitively. In this process, the questions focused on visual and acoustic feedback, since those feedback modularities are already widely used in medical devices and are the most suitable to use with proximal audio sensing.

Concerning visual feedback, the participants could choose from four different options:

1. Traffic light system via LEDs.
2. Simulation of an abdominal wall including a penetrating needle displayed on an external screen.
3. Display of force measurements.
4. Real-time plot of the audio signal.

**Fig. 6:**
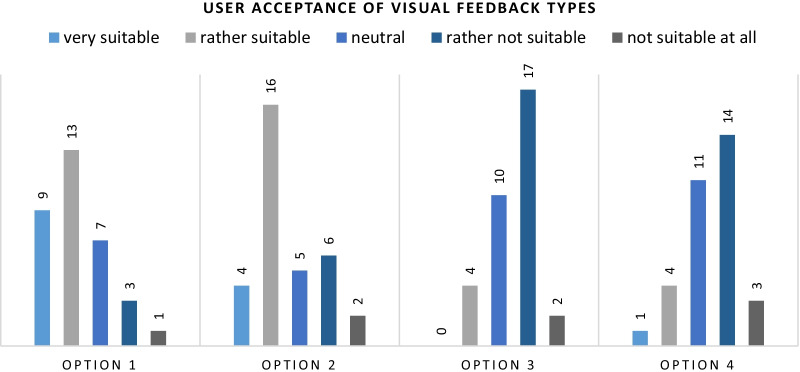
Most participants prefer visual feedback via LEDs integrated into the sensing module or on an external screen. Force measurements and real-time plots of the acquired audio signal were not accepted

The participants clearly voted in favor of the traffic light system (22 votes in the upper box) and the simulation displayed on an external screen (20 votes in the upper box). The reason for this could be that those two feedbacks are the simplest to understand and interpret. In contrast to that, the force measurement (19 votes in the lower box) will not impart any meaningful information to a person not experienced with force measurements and trained in estimating the force they apply in the measure that is displayed (e.g., Newton [N]). Plots of the audio signal (17 votes in the lower box) would also require surgeons to train on interpreting them correctly to be able to draw valuable conclusions from them. An effort that most participants would not like to spend. The results are also displayed in Fig. 6.

The options presented for the acoustic feedback were the following three:

1. Beep-tone comparable to a park distance control.
2. Magnified, processed sound based on the acquired audio signal.
3. Verbal feedback, comparable to a GPS navigation system.

**Fig. 7:**
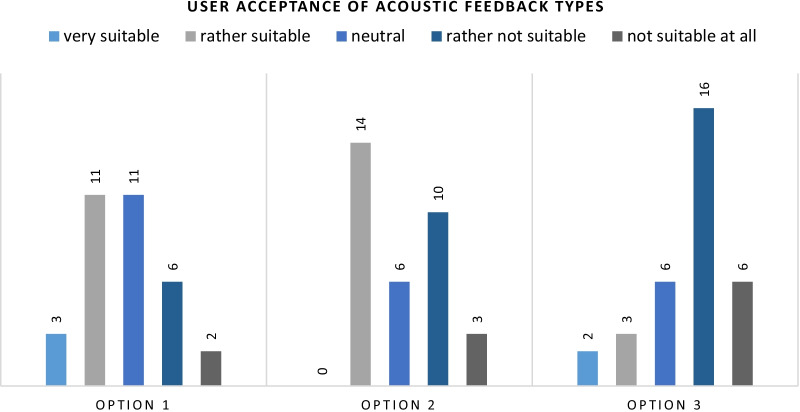
Verbal feedback during needle insertion was clearly rejected by the participants (66% in the lower box). Fourteen participants each found the acoustic feedback similar to a park distance control (option 1) and the continuous, magnified audio signal (option 2) suitable. However, option 1 was only rejected by eight participants in the lower box, while 13 participants rejected option 2

The acceptance rates of those variants are displayed in Fig. 7.

The navigation system like verbal feedback was clearly rejected by the participants with 22 votes in the lower box. The magnified audio signal (option 2) showed a divided response. Fourteen participants indicated that this approach is suitable, while 13 stated it was not. Six participants chose the neutral option. This might be caused by the fact, that this feedback approach is hard to imagine if not demonstrated live. The acoustic feedback inspired by a park distance control achieved the best result. Fourteen participants found it suitable, but only eight found it not suitable to impart feedback during laparoscopic access. Eleven remained undecided.

In summary, most participants preferred the feedback options that are already familiar to them (traffic light system, park distance control), that are, therefore, easy to perceive and process as well as impart specific information that do not require a lot of interpretation by the users.

## Discussion and conclusion

The creation of the capnoperitoneum during laparoscopic access is a critical step in the beginning of each laparoscopic surgery. The Veress needle is commonly used for this procedure to insufflate CO_2_ into the peritoneal cavity. However, since there is no support system for surgeons during this step, they have to rely solely on their tactile perception and practical experience to place the needle safely, leading to complication rates of up to 14%. Alternative methods such as optical trocars have been developed to improve patient safety but could not prove any advantage (see "Introduction" section) and have not been widely adopted by the clinical practitioners.

This explorative study was conducted to gather more insight into the problematic from the surgeons’ perspective, with the goal to include such information into the development of a surgical support system that could make laparoscopic access more precise, effective and safer. For this purpose, semi-structured interviews were conducted with surgeons from different career levels who are experienced in laparoscopic access. The insights were used to develop an online questionnaire which was distributed to surgeons in Germany, Austria and Switzerland.

The findings confirm that laparoscopic access with the Veress needle is a problematic step in laparoscopic surgery. Especially, the participating young surgeons indicated to feel uneasy and perceive a risk for punctures when piercing with the Veress needle. A fact that was not confirmed by surgeons using alternative tools like trocars, who also perceive less risk during insertion. However, participants stated that the effort for preparation and insertion is lower in the average case with the Veress needle. Additionally, most surgeons stated to use the Veress needle or the alternative tool respectively, because they were trained on it during their early career or because it is the commonly used method at their clinic. This indicates that, despite the Veress needle’s safety issues, surgeons potentially still use it, because they are used to it. Consequently, a surgical support system for laparoscopic access should allow the surgeons to continue using the tool they are familiar with.

On the other hand, most surgeons stated that the feedback of the Veress needle’s spring mechanism, which is used by them to guide the instrument, is not clearly perceivable and specific enough. This highlights a major problem of the Veress needle: the main goal of the procedure, the placement of the needle in the peritoneal cavity, is not clearly and reliably recognizable by the surgeons. In consequence, they can never be certain if they reached the peritoneal cavity. Indirect tests like the hanging drop test or the insufflation of the CO_2_ are time consuming and not always reliable.

Further, the participating users of both instruments indicated to rely on practical experience and tactile feedback, confirming previous findings in the scientific literature. In relation to the fact, that all participating residents, who have less experience, stated to feel uneasy during Veress needle insertion, it can be assumed that there could be a correlation between experience and complications during laparoscopic access. That such correlations exist was confirmed by the literature: A systematic review analyzing 51 studies found a relation between surgical experience and surgical performance [32].

The participants stated that two key information are required for performing laparoscopic access precise and safe: the information that the peritoneal cavity was reached as well as an alarm if an intraabdominal structure has been potentially injured. That information can neither be provided by the Veress needle, nor by alternative methods like trocars, for which most participants stated to also rely on practical experience and tactile perception.

According to this study’s participants, such information should be provided as using a traffic light system, preferably displayed via LEDs or a small display within their working focus. Displaying the information using a simulation of the abdomen with a proceeding needle might be acceptable. Acoustic feedback in general was rejected by most participants, which could be due to the already high noise levels in operating rooms [33]. An exception was the acoustic feedback that is displayed via a speaker that is integrated into the sensing module, for which the participants were divided (15 approvals, 14 rejections, 4 neutral). Wearable devices such as AR-glasses or headsets were consistently rejected, so these devices are not an option, at least, for short procedures such as laparoscopic access. While this study was tailored to laparoscopic access, these findings can also be applied to many other minimally invasive procedures.

In general, participants’ acceptance for discrete feedback variants (e.g., traffic light system and park distance control) was higher than for continuous feedback variants. However, it also needs to be considered, that the participants are already familiar with traffic light systems and park distance controls, which could have influenced their decisions. Moreover, continuous feedback variants are hard to explain in text, and should rather be demonstrated with prototypes to gather realistic information about their acceptance among surgeons.

The participants of this study were mainly active in the area of general surgery (17 participants) and urology (9 participants). While three participants practice gynecology, none of them was a pediatric surgeon. This could influence some results, since pediatric surgeons might perceive laparoscopic access as a more significant problem than surgeons performing it on adults. This is due to anatomic and size constraints in children, such as a more intraperitoneal position of the bladder and the closer proximity of the great vessels [34, 35]. The study’s results regarding the problematic of laparoscopic access also need to be put into context with the existing underreporting of complications during laparoscopic access (see "Introduction" section). Additionally, all participants of the study were practicing in Germany and Austria and, therefore, might not be representative for the situation in other countries. Although the number of participants was limited, this article contributes important insights to the research field, as no comparable articles have been published previously. Nevertheless, the results should be confirmed by a study with more participants.

Twenty-six out of 33 participants reported to perform laparoscopic access multiple times per week. In consequence, the study’s population was quite experienced, which might not be consistent with the average population of surgeons performing laparoscopic access. As the findings indicate that young surgeons with less experience are more probable to feel uneasy and perceive higher risk during laparoscopic access, this might also hold true for older surgeons who perform laparoscopic access less frequently, e.g., in rural areas. Those surgeons might have a higher demand for a surgical support system for laparoscopic access. If such a system could be established in clinical practice, it might lead to a more even quality of care among all kinds of clinics.

In summary, according to the findings of this study a surgical support system for laparoscopic access should provide information about the reaching of the peritoneal cavity and indicate to the surgeon if an intraabdominal structure was hit and potentially injured. Such information should be presented as visual or acoustic feedback, and it should be evaluated if a combination of both could have advantages for the overall population of surgeons. Visual feedback should be presented via LEDs or an external screen, while the acoustic feedback could be similar to a park distance control. Some participants would also accept acoustic feedback that consists of the acquired audio signal, which is processed, magnified and then played via speakers. This feedback variant might be hard to imagine for some surgeons, so prototypes should be developed and tested with an intermixed population of surgeons. Finally, during the semi-structured interviews prior to the online questionnaire, many surgeons indicated that current solutions are expensive, complex to use and negatively impact the clinical workflow—which results in most surgeons not using such systems. To avoid this, a surgical support system for laparoscopic access should be very simple and intuitive to use, should support or even improve the clinical workflow, but also be cheap enough to facilitate its usage rate. The features that a surgical support system for laparoscopic access should provide are summarized in Table 2.

**Table 2:** The main features of a surgical support system for laparoscopic access and the preferred configuration according to the study participants

Criteria	Participants’ choice
Required information	Reaching of peritoneal cavity AND Injury of an intraabdominal structure
Feedback modality	Visual OR Acoustic
Visual feedback	LEDs (Traffic Light System)
Acoustic feedback	Similar to Park Distance Control
Usage	Simple, intuitive, quick
Workflow	Minor or no changes

## Supplementary Information

**Additional file 1:** Survey results as raw data.

## Data Availability

All data generated or analyzed during this study are included in this published article and its additional information files.
